# Differences in the individual curative effect of acupuncture for obese women with polycystic ovary syndrome based on metagenomic analysis: study protocol for a randomized controlled trial

**DOI:** 10.1186/s13063-021-05426-y

**Published:** 2021-07-15

**Authors:** Huaying Fan, Xiaojuan Hong, Jiuzhi Zeng, Xue Wang, Jiao Chen

**Affiliations:** 1 School of Acupuncture-Moxibustion and Tuina/The Third Affiliated Hospital, Chengdu University of Traditional Chinese Medicine, Chengdu, China; 2 Department of Reproductive Medicine, Sichuan Women’s and Children’s Hospital, Chengdu, China

**Keywords:** Acupuncture, Obesity, Polycystic ovary syndrome, Gut microbiota, Protocol

## Abstract

**Background:**

Polycystic ovary syndrome is a common cause of infertility and shows a high incidence in women of reproductive age. Acupuncture is an appropriate adjunctive treatment for PCOS. However, the add-on effect of acupuncture as an adjunctive treatment for obese women with PCOS has not been studied, and previous studies indicate that there are individual differences in the curative effect of acupuncture, while deeper research on the mechanism of differences in the individual curative effect of acupuncture for obese women with PCOS is still lacking. This trial aims to assess the add-on treatment efficacy of acupuncture for obese women with PCOS and to explore the role of the gut microbiome on the differences in the individual curative effect of acupuncture based on metagenomic analysis.

**Methods/design:**

This is an open-label, randomized, controlled trial. A total of 86 obese women with PCOS will be recruited. Subjects will be randomly assigned to a study group and a control group in a 1:1 ratio, with 43 subjects in each group (10 patients from each group who meet the study criteria will participate in the metagenomic analysis). An additional 10 subjects who meet the study criteria will be recruited to a healthy control group. The study group will receive acupuncture and clomiphene citrate treatment; the control group will only receive clomiphene citrate. Acupuncture treatment will be conducted three times a week from the fifth day of menstruation or withdrawal bleeding until the start of the next menstruation, for up to three menstrual cycles. The primary outcome will be LH/FSH. The secondary outcomes will comprise biometric features, hormone biomarkers, metabolic biomarkers, inflammatory biomarkers, Self-Rating Anxiety Scale, Self-Rating Depression Scale, and metagenomic analysis. The outcomes will be measured at baseline and post-intervention. Data will be analyzed using SPSS 19.0, and the gut microbiome will be analyzed using metagenomic analysis.

**Discussion:**

In this study, we are evaluating the add-on effects of acupuncture and exploring the mechanism of the differences in the individual curative effect of acupuncture based on the gut microbiome, which may provide evidence to explain the different outcomes of different trials on acupuncture for PCOS and hopefully to provide a new aspect to study the mechanism of acupuncture’s treatment effect.

**Trial registration:**

Chinese Clinical Trial Registry ChiCTR2000029882. Registered on 16 February 2020

**Supplementary Information:**

The online version contains supplementary material available at 10.1186/s13063-021-05426-y.

## Background

Polycystic ovary syndrome (PCOS) is a common endocrine and metabolic disorder characterized by androgen excess (hirsutism and/or hyperandrogenemia) and ovarian dysfunction (oligo-ovulation and/or polycystic ovarian morphology) [1]. The estimated prevalence of PCOS varies from 5 to 20% in women of reproductive age [2–8], and about 50% of PCOS cases are associated with obesity [9]. As one of the most common causes of subfertility, PCOS accounts for 30 to 40% of infertility and 75% of anovulatory infertility [10]. The increasing prevalence of obesity has an important bearing on the PCOS phenotype [11, 12], and women diagnosed with PCOS who have higher body mass index (BMI) are more likely to have menstrual irregularity, hyperandrogenemia, hirsutism, and insulin resistance [13–15], which can exacerbate the reproductive and metabolic abnormalities of PCOS [16]. Obese women with PCOS are more likely to experience psychological disorders, such as depression and anxiety [17], and babies born to women with PCOS may have increased morbidity and mortality [15], which can be a big burden for both patients and the healthcare system.

Clomiphene citrate, as the first-line ovulation induction drug, has been used among infertile PCOS women with fertility desires for decades. However, from a clinical point of view, the current management of PCOS with this drug is not always satisfactory and produces some obvious side effects [18]. After taking clomiphene citrate, 39.22% of women still failed to ovulate. The live birth rate for these patients was only 7.84% after taking clomiphene citrate. Other side effects include gastrointestinal symptoms, hot flashes, headache, fatigue, abdominal cramping, and symptoms associated with ovarian enlargement and ovulation [19, 20]. Thus, an increasing number of patients with PCOS are seeking complementary and alternative medicine (CAM) methods such as acupuncture for fertility assistance [21, 22].

Acupuncture, as a part of CAM, has been long used to treat PCOS. Although researches have shown that acupuncture can activate the neural-endocrine-immune network and increase beta-endorphin production to balance the endocrine system and hormones, as well as affect ovulation and menstrual cycle, evidence for acupuncture’s utility for PCOS is conflicting [23–30]. Findings from previous clinical studies suggest acupuncture may induce ovulation [27], improve menstrual frequency, and decrease the levels of several sex steroids [28]. Additionally, the low associated adverse event rate, a low risk of multiple pregnancies, and the low cost of acupuncture have been noted [31]. Nevertheless, the methodological quality of these clinical trials varies, resulting in inconclusive statements on the effectiveness of acupuncture for PCOS. Meanwhile, the add-on effect of acupuncture as an adjunctive treatment for obese women with PCOS has not been studied, although acupuncture has shown some efficacy in managing PCOS, especially in conjunction with pharmaceutical treatments [26].

Furthermore, a study found a higher ovulation rate in the active acupuncture group than in the clomiphene alone group, but the results indicated that acupuncture did not affect the live birth rate [32]. Another study also found an improvement in the ratio of luteinizing hormone (LH) to follicle-stimulating hormone (FSH) during the 8 weeks of intervention, and this persisted for 3 months of follow-up in the acupuncture arm; however, there was no difference in ovulation rate between the true acupuncture group and the sham acupuncture group over 5 months [33]. Given these contrasting findings, we hypothesize that the curative effect of acupuncture for PCOS is associated with individual differences, that is, whether patients show sensitivity to acupuncture is one of the differences in the individual curative effect of acupuncture. However, the cause of differences in the individual curative effect of acupuncture for obese women with PCOS has not yet been studied.

Recently, there has been an explosion of interest in the study of the gut microbiome and its impact on host health and physiology [34]. The gut microbiome comprises the collective genome of the trillions of microorganisms residing in our gastrointestinal ecosystem [35]. The composition and activity of the gut microbiome have a substantial effect on human health and are regarded as the main cause of individual differences [36]. Studies have shown that the gut microbiome is strongly associated with PCOS, as well as the development and clinical parameters of PCOS. Studies have pointed out that the stool microbiome of PCOS patients shows a lower alpha diversity [37–41]; the relative abundance of bacteria from the phylum Tenericutes, the order ML615J-28 (phylum Tenericutes), and the family S24-7 (phylum Bacteroidetes) is significantly lower than health controls [37]. Besides, researches indicate that the gut microbiota-bile acid interleukin-22 axis or the brain-gut axis may be the potential mechanism in the development progress of PCOS, and the dysbiosis of the gut microbiota is strongly relevant to endocrine disorders, including LH, FSH, LH/FSH, anti-Mullerian hormone (AMH), and testosterone, in PCOS patients [38, 42–44]. Of note, the gut microbiome also plays an important role in the curative effect of acupuncture. Acupuncture research has demonstrated that acupuncture can regulate the brain-gut-bacteria axis and reverse disturbances in the gut microbiota metabolism [45, 46]. Electro-acupuncture treatment can normalize several chronic atrophic gastritis (CAG)-induced metabolomic changes by partially reversing CAG-induced disturbances in the gut microbiota metabolism [46]. As gut microbiota is a major target for diseases’ treatment and may predict responses to clinical therapy [47], as well as it is strongly associated with PCOS and the curative effect of acupuncture, thus, we speculate that acupuncture might play a therapeutic role in women with PCOS by regulating the gut microbiome, and the gut microbiome may be strongly associated with the differences in the individual curative effect of acupuncture for obese women with PCOS.

Considering that the add-on effect of acupuncture as an adjunctive treatment for obese women with PCOS has not been studied, and acupuncture for obese women with PCOS may exist individual curative effect differences, we designed this randomized controlled trial (RCT) to evaluate the add-on effects of acupuncture to conventional drugs in obese women with PCOS, as well as to investigate the mechanism of differences in the individual curative effect of acupuncture for obese women with PCOS by using metagenomic analysis method.

## Methods/design

### Study design

This will be an open-label, randomized, controlled trial. The study will recruit 86 obese patients with PCOS from Sichuan Women’s and Children’s Hospital, Sichuan Provincial Hospital of Traditional Chinese Medicine, the Third Affiliated Hospital of Chengdu University of Traditional Chinese Medicine, or Xinan Women’s and Children’s Hospital. The patients enrolled will be randomly assigned to a study group or a control group. The allocation ratio is 1:1, with 43 subjects in each group. All patients involved in this trial will be asked to sign the consent form before the intervention by researchers. Patients in the study group will be given acupuncture and clomiphene citrate treatment, and the control group will receive only clomiphene citrate treatment.

**Fig. 1:**
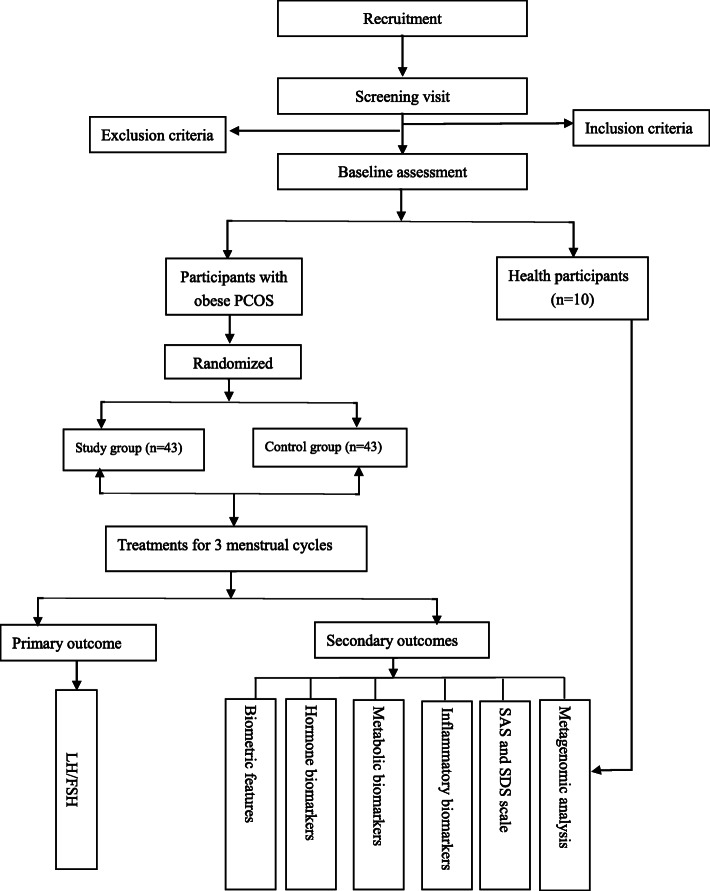
Study flow chart. Note: metagenomic analysis includes 10 patients in the study group and the control group separately. Among the 10 patients in each group, 5 patients with the serum LH/FSH level among 1.0–1.5 after the intervention and 5 patients with the serum LH/FSH level over 1.5

In addition, with the purpose to explore the mechanism of the differences in the individual curative effect of acupuncture, 10 subjects from each group will be selected in the order of enrollment for the metagenomic analysis. The selection criteria for these 10 subjects from each group will be that 5 of them with post-intervention LH/FSH levels of 1.0–1.5 and the other 5 subjects with post-intervention LH/FSH levels over 1.5. Meanwhile, ten healthy subjects who meet the study criteria will be recruited as the healthy control group and will not be given any intervention. The flow chart of the trial process is shown in Fig. 1. The research data collection is described in detail in Table 1.

**Table 1:** Overview of study visits

Item	Screening visit	Baseline visit	Treatment visit		End of treatment visit
			Study group	Control group	
Sign consent	**×**				
History	**×**				**×**
Biometric features	**×**	**×**			**×**
Transvaginal ultrasound	**×**	**×**	**×**	**×**	**×**
Routine test	**×**				**×**
Hormone biomarkers		**×**			**×**
Metabolic biomarkers		**×**			**×**
Inflammatory biomarkers		**×**			**×**
Metagenomic analysis		**×**			**×**
Questionnaires		**×**			**×**
Acupuncture treatment			**×**		
Adverse events			**×**	**×**	

*Note:* Participants from the healthy control group will be selected for metagenomic analysis and will only be assessed at the initial screening visit. History, biometric features, routine test results, and metagenomic analysis will be assessed. Biometric features include body mass index (BMI) and waist circumference; transvaginal ultrasound includes endometrial thickness, ovarian volume, antral follicle count, and size of ovarian cysts or developing follicles; Routine tests include blood routine examination, liver function, kidney function, routine stool, and urine tests; Hormone biomarkers include luteinizing hormone (FSH), follicle-stimulating hormone (LH), total testosterone, progesterone, estradiol, and leptin; metabolic biomarkers include fasting blood glucose (FBG), fasting insulin (FINS), homeostatic model assessment for insulin resistance (HOMA-IR), c-peptide release tests, total cholesterol (TC), triglycerides (TG), high-density lipoprotein (HDL-C), and low-density lipoprotein (LDL-C); inflammatory biomarkers include C-reactive protein, interleukin-6, interleukin-18, and interleukin-22; questionnaires include Self-Rating Anxiety Scale (SAS) and Self-Rating Depression Scale (SDS)

This study protocol is compliant with the principles of the Consolidated Standards of Reporting Trials (CONSORT) guidelines and the Standards for Reporting Interventions in Clinical Trials of Acupuncture (STRICTA) [48, 49], as well as with the Standard Protocol Items: Recommendations for Intervention Trials (SPIRIT) [50]. The SPIRIT checklist is presented in Additional file 1.

### Participants

#### Recruitment

Participants will be recruited from the gynecological outpatient departments of Sichuan Women’s and Children’s Hospital, Sichuan Provincial Hospital of Traditional Chinese Medicine, the Third Affiliated Hospital of Chengdu University of Traditional Chinese Medicine, or Xinan Women’s and Children’s Hospital. Recruitment posters, advertisements, and internet advertisements will be used to recruit participants. Before participation, patients will be informed about the study design, the advantages and disadvantages of the treatment, and the relevant safety measures that will be used during the trial.

#### Inclusion criteria

##### Inclusion criteria for PCOS

PCOS diagnosis will be based on the *Revised 2003 Consensus on Diagnostic Criteria and Long-Term Health Risks related to Polycystic Ovary Syndrome* established by the Rotterdam ESHRE/ASRM-Sponsored PCOS Consensus Workshop Group [51]. According to the criteria, women with two or three of the following symptoms can be diagnosed with PCOS: hyperandrogenism, ovulatory dysfunction (oligo-ovulation or anovulation), and polycystic ovary morphology based on ultrasound.

The inclusion criteria are as follows: (1) infertile female with reproductive needs aged 20 to 40 years, (2) meets the diagnostic criteria for PCOS, (3) BMI ≥ 25 kg/m^2^, (4) suitable for administration of clomiphene as an ovulation induction treatment, and (5) has signed an informed consent form.

##### Inclusion criteria for healthy participants

The inclusion criteria for healthy participants are as follows: (1) female patient aged 20 to 40 years, (2) BMI ≥ 18.5 kg/m^2^ and ≤ 25 kg/m^2^, (3) with regular menstruation, (4) no primary dysmenorrhea history in the past years, (5) normal blood/stool/urine test, (6) no mental illness, and (7) has signed an informed consent form.

#### Exclusion criteria

##### Exclusion criteria for PCOS

Subjects will be excluded if they have any of following conditions: (1) patient does not meet the diagnostic criteria for PCOS; (2) with history of taking antibiotics in the past 3 months; (3) with allergic condition; (4) with conditions such as hyperandrogenism due to hyperprolactinemia, thyroid disease, congenital adrenal hyperplasia, and Cushing syndrome; (5) with pathological endometrial changes, such as uterine malformation and hysteromyoma diagnosed using ultrasound; (6) with conditions such as genital tract malformation, gonadal dysgenesis, and fallopian tube blockage; (7) with severe heart, liver, or renal dysfunction; or hematological, respiratory, cardiovascular, and psychiatric; (8) patient has participated in other clinical trials; (9) patients with gastrointestinal surgery history; (10) patient has smoking history, alcohol use history, inflammatory bowel disease, irritable bowel syndrome, autoimmune diseases, cancer, or any other diseases that may influence the gut microbiome; (11) patient has probiotics taking history within 1 month; (12) with uncontrolled disease(s) that cause inflammation; and (13) patient has diagnosed with anxiety or depression.

Items from (1) to (8) are suitable for clinical observation. Items from (1) to (13) are suitable for metagenomic analysis.

##### Exclusion criteria for healthy participants

Healthy participants with any of the following conditions will be excluded from this study: (1) with antibiotics taking history in the past 3 months; (2) with smoking history and/or alcohol use; (3) with mental disorders, cognitive disorders, or allergic conditions; (4) patient is pregnant or lactating; (5) with gastrointestinal surgery history; (6) with inflammatory bowel disease, irritable bowel syndrome, autoimmune diseases, cancer, or other diseases that may influence the gut microbiome; (7) with probiotics taking history within 1 month; (8) with uncontrolled disease(s) that cause inflammation; and (9) patient has participated in other clinical trials.

### Intervention

All participants will be informed of the benefits of healthy lifestyle habits, such as regular exercise and healthy eating. The study group will receive acupuncture and clomiphene citrate treatment; the control group will receive only clomiphene citrate. The intervention in both groups will last three menstrual cycles. Acupuncture treatments will be given by professional acupuncturists with at least 2 years of practical acupuncture experience.

#### Acupuncture treatment

Acupuncture points will be selected based on the Zang-fu organ system, Yin-Yang theory, and clinical rules for PCOS acupoint selection. Two sets of acupoints will be used; each set will be used on alternate treatments. The first acupoint formula comprises DU-20, DU-24, GB-13, RN-12, ST-25, RN-4, EX-CA-1, KI-12, SP-6, and LR-3. The second acupoint formula comprises BL-23, BL-32, SP-6, and KI-3. Disposable, single-use, sterilized needles (Huatuo, Suzhou Medical Appliance Fact. 215005 Suzhou, China) of sizes 0.25 mm × 25 mm, 0.25 mm × 40 mm, and 0.25 mm × 50 mm will be inserted into the acupoints, and a *Deqi* sensation obtained by manipulating the needles. Table 2 shows the location, depth, and type of insertion for each acupoint. Each treatment will last for 30 min; no manipulation will be used once the *Deqi* sensation is achieved. The acupuncture treatment will be conducted three times a week from the fifth day of menstruation or withdrawal bleeding until the start of the next menstruation, for up to three menstrual cycles.

**Table 2:** Acupuncture protocol

Acupoints	Location	Method
**Set 1**		
DU-20 (Baihui)	On the head, 5*cun* directly above the midpoint of the anterior hairline	0.5–1.0*cun* subcutaneous insertion
DU-24 (Shenting)	On the head, 0.5*cun* directly above the midpoint of the anterior hairline	0.5–1.0*cun* subcutaneous insertion
GB-13 (bilateral) (Benshen)	On the head, 0.5*cun* within the front hairline, 3*cun* from Shenting (GV-24)	0.5–1.0*cun* subcutaneous insertion
RN-12 (Zhongwan)	On the anterior median line of the upper abdomen, 4*cun* below the navel	1.0–1.5*cun* perpendicularly insertion
ST-25 (bilateral) (Tianshu)	On the middle portion of the abdomen, 2*cun* lateral to the center of the navel	1.0–1.5*cun* perpendicularly insertion
RN-4 (Guanyuan)	On the anterior median line of the lower abdomen, 3*cun* below the navel	1.0–1.5*cun* perpendicularly insertion
EX-CA-1 (bilateral) (Zigong)	On the lower abdomen, 4*cun* below the navel, 3*cun* lateral to Zhongji (RN-3)	1.0–1.5*cun* perpendicularly insertion
KI-12 (bilateral) (Dahe)	On the lower abdomen, 4*cun* below the center of the navel, 0.5*cun* lateral to the anterior midline	1.0–1.5*cun* perpendicularly insertion
SP-6 (bilateral) (Sanyinjiao)	On the medial side of the shank, 3*cun* above the medial malleolus, by the posterior of the tibia	1.0–1.5*cun* perpendicularly insertion
LR-3 (bilateral) (Taichong)	On the dorsum of the foot, in the depression proximal to the first metatarsal space	1.0–1.5*cun* perpendicularly insertion
**Set 2**		
BL-23 (bilateral) (Shenshu)	On the lower back, 1.5*cun* lateral to the lower border of the spinous process of the 2nd lumber vertebra	0.5–1.0*cun* perpendicularly insertion
BL-32 (bilateral) (Ciliao)	In the region of the sacrum, between the posterior superior iliac spine and the posterior median line, in the 2nd posterior sacral foramen	2.5–3.0*cun* oblique insertion to the 2nd posterior sacral foramen
SP-6 (bilateral) (Sanyinjiao)	On the medial side of the shank, 3*cun* above the medial malleolus, by the posterior of the tibia	1.0–1.5*cun* perpendicularly insertion
KI-3 (bilateral) (Taixi)	On the medial side of the foot, posterior to the medial malleolus, in the depression between the tip of the medial malleolus and the tendon calcaneus	0.5–1.0*cun* perpendicularly insertion

*Note*: Two sets of acupoints will be used; each set will be used on alternate treatments

#### Clomiphene citrate treatment

Clomiphene citrate treatment will be administered on the fifth day of menstruation to participants without amenorrhea in the study and control groups. Participants will receive treatment with an initial oral dose of 50 mg for 5 continuous days. If there is an ovulatory response, this dose will be maintained and will be given on the next cycle. For patients with no ovulatory response, an additional 50 mg dose will be given on the next cycle; the maximum dose of clomiphene citrate will not exceed 150 mg. Patients with amenorrhea will receive the same pharmacological treatment regimen after withdrawal bleeding has been induced by progestin. The treatment will last for three menstrual cycles.

### Outcome measures

#### Primary outcome

The primary outcome will be the change of LH/FSH. Data will be collected before the start of the study (the baseline assessment) and after 3 menstrual cycle treatments.

#### Secondary outcomes

The secondary outcomes will include changes in biometric features, changes in hormonal parameters/metabolic parameters/inflammatory parameters, changes in Self-Rating Anxiety Scale (SAS) and Self-Rating Depression Scale (SDS) scores, and changes in gut microbiome analysis. Data will be collected before the start of the study and after 3 menstrual cycle treatments.

Biometric features related to obesity, including BMI and waist will be assessed. BMI will be calculated in kg/m^2^. Waist circumference will be measured as the smallest circumference between the lowest rib and the iliac crest [52, 53]. Blood samples to assess hormonal parameters, metabolic parameters, and inflammatory parameters will be collected in the morning after an overnight fast, on day 3 of a menstrual cycle. Sex hormonal parameters will include FSH, LH, total testosterone, progesterone, and estradiol. The adipocyte-secreted hormone will be assessed by leptin, which was discovered in 1994 and can cause abnormal energy homeostasis and profound obesity [54, 55]. Leptin circulates primarily at levels proportional to the amount of adipose tissue, signaling long-term energy storage, and secondarily at levels modified by acute changes in caloric intake [56]. Leptin should decrease weight when circulating at high levels [57], and it plays a clear role in obesity etiology [58]. The metabolic parameters will include fasting blood glucose (FBG), fasting insulin (FINS), homeostatic model assessment for insulin resistance (HOMA-IR), c-peptide release tests, total cholesterol (TC), triglycerides (TG), high-density lipoprotein (HDL-C), and low-density lipoprotein (LDL-C). HOMA-IR will be calculated using the following formula: FBG (mmol/L) × FINS (mIU/L)/22.5 [59]. Inflammatory parameters will include C-reactive protein (CRP), interleukin-6 (IL-6), interleukin-18 (IL-18), and interleukin-22 (IL-22). These cytokines are related to inflammatory cytokines involved in PCOS [60–62]. SAS and SDS scores will be used to assess the patients’ mental health. The Self-Rating Anxiety Scale (SAS) and Self-Rating Depression Scale (SDS) can discriminate anxiety and depression from mood disorder and have been used continuously in research since 1972 [63, 64].

To explore the role of the gut microbiome in individual curative effect differences of acupuncture, a metagenome-wide association study (MWAS) method will be used. MWAS is a powerful tool with which to explore the microbiome and enables high-resolution investigation of associations between the human microbiome and some complex diseases. These associations are not limited to the identification of more or less abundant taxa, but also include the identification of enriched or depleted microbial functions [65]. Before the stool samples are collected, a patient involved in the metagenomic analysis will be asked to fill out a Diet and Lifestyle Habits Questionnaire. Fresh fecal samples will be collected from the participants in the morning after an overnight fast on their first visit and the end of intervention using empty stool collection tubes with an inbuilt spatula (Praxisidienst GmbH, Longuich, Germany). Their samples will be then transported to the laboratory with an ice pack within 2 h. All samples then are frozen and stored at − 80 °C before the analyses. The metagenomic analysis will be performed by BGI Shenzhen Co., Ltd. (China).

The metagenomic analysis will include 6 parts: (1) operational taxonomic units (OTUs): comparison the composition of intestinal microbiota at the level of phyla, classes, orders, families, genera, and species in each group; (2) alpha diversity: comparison the richness of species (the Chao 1 index), the abundance of the species (the Shannon-Wiener index and the Simpson index), and the evenness of the species (the Shannoneven index and the Simpsoneven index) in each group; (3) beta diversity: principal component analysis (PCA) and principal coordinate analysis (PCoA) will be used to identify the beta diversity in each group and hopefully to understand whether the differences in the microbiota compositions of groups are significant; (4) the taxonomic profile: comparison of the variety and relative abundance of taxon in the study group and control group at the level of phyla, classes, orders, families, genera, and species; (5) gene abundance profile: in order to identify the specific genes related to the individual curative effect differences of acupuncture, gene abundance will be profiled between the study group and control group, as well as within group (5 patients with post-intervention serum LH/FSH levels of 1.0–1.5 and 5 patients with serum LH/FSH levels over 1.5); (6) metagenomic biological pathways: selecting genes annotated by Kyoto Encyclopedia of Genes and Genomes (KEGG) to evaluate the functional aspects of the PCOS gut metagenome and hopefully to identify the metagenomic biological pathways contributing to the mechanism of individual curative effect differences of acupuncture for obese women with PCOS.

### Adverse events

Adverse events of acupuncture mainly involve local ecchymoses, fainting, serious pain, and local infection, with close relevance to the subjective experience of patients and the penetration technique of acupuncturists. All the acupuncturists will undergo pretrial training and an entrance examination before the trial to ensure a safe operation. We will record any acupuncture-related adverse events that occur during the treatment, and operators will take necessary corresponding treatment measurements. In case of serious adverse events, the clinical trial will be interrupted immediately by investigators, and an effective treatment shall be taken. At the same time, the serious adverse events will be reported to the ethical review board in the hospital of Chengdu University of Traditional Chinese Medicine within 24 h. The whole process will be recorded in detail.

### Sample size

A previous study has found an average LH/FSH ratio for patients with PCOS after true acupuncture treatment of 1.4 [33]. Thus, we expect that the average LH/FSH ratio will be 1.0 in the study group and 1.6 in the control group. Assuming a power of 80% and a significance level of 0.05, 37 participants in each group will be required, as calculated using the *t*-test in G*Power. Based on a 15% dropout rate, 86 patients will be recruited, with no less than 43 participants in each group. Since there is no standard sample size calculation for metagenomic analysis at present, according to most of the studies about gut microbial studies for PCOS [44, 66], of the 86 participants, 10 patients in each group (5 patients with post-intervention serum LH/FSH levels of 1.0–1.5 and 5 patients with serum LH/FSH levels over 1.5) will be selected for metagenomic analysis.

### Randomization and allocation concealment

Patients will be randomly assigned to the study group or control group in a 1:1 ratio using a random number table. The random numbers will be provided by an independent statistician using the SPSS statistical software (version 19.0, SPSS Inc., Chicago, IL, USA). Random numbers and treatment protocols will be placed in sealed envelopes by an independent investigator. When a patient is enrolled, acupuncturists will check whether the envelope is sealed and then open the envelope to find the random number and treatment protocol for the patient.

### Blinding

As this is an open-label trial, neither the patients nor the acupuncturists will be blinded for the treatment. However, to eliminate potential bias, other researchers (including data collectors and statisticians) will be blinded.

### Data collection and management

Data collection will be conducted at baseline and post-intervention. Data collectors will record participant data on case report forms (CRFs). All data collectors will receive training on how to administer the SAS and SDS scales. After data collection using CRFs, we will use an electronic database for data entry and management. Two data collectors will perform double data entry, and completed clinical raw data will be forwarded to the supervisor of the project for review. The supervisor will correct any errors and export the data. After confirmation, the database will be locked and undergo statistical analysis. All data will be identified using participant numbers and stored in the database, which will be accessed using a password. The data will be monitored by the ethical review board of the Hospital of Chengdu University of Traditional Chinese Medicine.

The incidence of withdrawal from the study and the withdrawal reasons will be reported. Dropouts will be included in the analysis using modern imputation methods for missing data.

### Statistical methods

Data will be analyzed using SPSS version 19.0 (SPSS 19.0, SPSS Inc., Chicago, IL, USA) by two qualified statisticians. Full analysis set (FAS), per-protocol analysis set (PPS), and safety set (SS) principles will be used to evaluate the final results. FAS is determined according to an intention-to-treat (ITT) population and includes all patients who have received at least one treatment. PPS will include all patients with good compliance who have completed the entire intervention. SS will include all patients who have received at least one intervention. The results of the measurement data analysis will be described using mean, median, 25th percentile, 75th percentile, standard deviation, maximum values, and minimum values. The comparison between the groups will be performed using the independent sample *t*-test or the Mann-Whitney *U* rank sum test according to whether the measurement data show a normal distribution and homogeneous variance. Frequency and constituent ratios will be used to describe enumeration data, and Pearson’s *χ*^2^ test or Fisher’s exact test will be used for group comparisons. Pearson correlations and Spearman correlations will be used to analyze the correlations between different indicators. All statistical tests will be considered statistically significant at *P <* 0.05 with a two-sided test. The gut microbiome will be analyzed using metagenomic analysis by BGI Shenzhen Co., Ltd (China).

### Patient and public involvement

Patients were not involved in the design and conduct of the study. Participants will be able to view the study results via social media.

## Discussion

PCOS is a complex reproductive disorder with multiple manifestations. Women with PCOS are more likely to be overweight [67], and excess weight worsens the features of PCOS [68]. Then, weight management plays an important role in PCOS [69]. Acupuncture is an acceptable adjunct to lifestyle interventions for weight loss in obese women with PCOS [30] and so could improve the treatment of PCOS. There is evidence that acupuncture can improve menstrual frequency and ovulation frequency, decrease the levels of several sex steroids (including testosterone, androsterone glucuronide, androstane-3α, and 17β-diol-3-glucuronide) in women with PCOS, and improve the LH/FSH ratio [27, 28, 33]. Although acupuncture seems to have a beneficial effect on PCOS, evidence for acupuncture’s utility for PCOS is conflicting [70], and the add-on effect of acupuncture for obese women the PCOS has not been studied. Besides, we speculated that the differences in the individual curative effect of acupuncture may explain the inconsistent results, and to the best of our knowledge, no studies have examined the potential mechanism of individual differences in acupuncture’s curative effect in obese women with PCOS. This will be the first trial to compare acupuncture with clomiphene citrate in obese women with PCOS with the aim of exploring the connection between acupuncture and the gut microbiome. On the basis of previous clinical studies and studies on the mechanism of acupuncture, we hypothesize that acupuncture will improve biomarkers and symptoms by regulating the gut microbiome composition of obese women with PCOS. Our findings will add to research on the PCOS phenotype and elucidate the role of the gut microbiome in individual curative effect differences of acupuncture based on metagenomic analysis.

There are three limitations to this study. First, participants’ stool may be influenced by many factors, even though strict inclusion and exclusion criteria have been made, so the research results may not accurately reflect the true mechanism of individual curative effect differences of acupuncture. Second, considering one of the aims of our study is to evaluate the add-on effect of acupuncture for obese women with PCOS, a sham acupuncture group is not assigned as the control group in this study, which could not exclude the placebo effect of acupuncture. Third, the sample size for metagenomic analysis is small compared to the clinical studies. As far as we know, although some methods for sample size and power calculations for microbiome research are used, such as *t*-test, analysis of variance, Dirichlet multinomial method, and *χ*^2^ test, the sample size for microbiome research still remains a challenge [71]. Effect size and statistical power are challenging to calculate in microbiome data, and applied power calculations typically make assumptions about the data that do not hold true in the analysis of microbial communities, as well as the power analysis remains rare in microbial studies [72]. In this study, we will include 10 patients in each group to do the metagenomic analysis. This might be a small sample size compared to the clinical studies, which may influence the differences among the groups, but considerations of cost and reimbursement and small size of sample size for microbial studies may be feasible [73].

### Trial status

The protocol version number is 20200115, V1.0 (2020/01/15). This study has started in September 2020, and the recruitment is expected to be completed in 2022.

## Supplementary Information

**Additional file 1.** SPIRIT Checklist.

## Data Availability

Data and materials can be obtained from the corresponding author after the trial.

## Abbreviations

PCOS: Polycystic ovary syndrome
BMI: Body mass index
CAM: Complementary and alternative medicine
LH: Luteinizing hormone
FSH: Follicle-stimulating hormone
AMH: Anti-Mullerian hormone
CAG: Chronic atrophic gastritis
RCT: Randomized controlled trial
CONSORT: Consolidated Standards of Reporting Trials
STRICTA: Standards for Reporting Interventions in Clinical Trials of Acupuncture
SPIRIT: Standard Protocol Items: Recommendations for Intervention Trials
FBG: Fasting blood glucose
FINS: Fasting insulin
HOMA-IR: Homeostatic model assessment for insulin resistance
TC: Total cholesterol
TG: Triglycerides
HDL-C: High-density lipoprotein
LDL-C: Low-density lipoprotein
CRP: C-reactive protein
IL: Interleukin
SAS: Self-Rating Anxiety Scale
SDS: Self-Rating Depression Scale
MWAS: Metagenome-wide association study
OTUs: Operational taxonomic units
PCA: Principal component analysis
PCoA: Principal coordinate analysis
KEGG: Kyoto Encyclopedia of Genes and Genomes
CRFs: Case report forms
FAS: Full analysis set
PPS: Per-protocol analysis set
SS: Safety set
ITT: Intention-to-treat
